# The putative prenyltransferase Nus1 is required for filamentation in the human fungal pathogen *Candida albicans*

**DOI:** 10.1093/g3journal/jkae124

**Published:** 2024-06-14

**Authors:** Aiman Farheen, Nicola T Case, Jessie MacAlpine, Ci Fu, Nicole Robbins, Leah E Cowen

**Affiliations:** Department of Molecular Genetics, University of Toronto, Toronto, ON M5G 1M1, Canada; Department of Molecular Genetics, University of Toronto, Toronto, ON M5G 1M1, Canada; Department of Molecular Genetics, University of Toronto, Toronto, ON M5G 1M1, Canada; Department of Molecular Genetics, University of Toronto, Toronto, ON M5G 1M1, Canada; Department of Molecular Genetics, University of Toronto, Toronto, ON M5G 1M1, Canada; Department of Molecular Genetics, University of Toronto, Toronto, ON M5G 1M1, Canada

**Keywords:** fungal pathogen, *Candida albicans*, filamentation, Rer2, lipid homeostasis

## Abstract

*Candida albicans* is a major fungal pathogen of humans that can cause serious systemic infections in vulnerable immunocompromised populations. One of its virulence attributes is its capacity to transition between yeast and filamentous morphologies, but our understanding of this process remains incomplete. Here, we analyzed data from a functional genomic screen performed with the *C. albicans* Gene Replacement And Conditional Expression collection to identify genes crucial for morphogenesis in host-relevant conditions. Through manual scoring of microscopy images coupled with analysis of each image using a deep learning-based method termed Candescence, we identified 307 genes important for filamentation in tissue culture medium at 37°C with 5% CO_2_. One such factor was *orf19.5963*, which is predicted to encode the prenyltransferase Nus1 based on sequence homology to *Saccharomyces cerevisiae*. We further showed that Nus1 and its predicted interacting partner Rer2 are important for filamentation in multiple liquid filament-inducing conditions as well as for wrinkly colony formation on solid agar. Finally, we highlight that Nus1 and Rer2 likely govern *C. albicans* morphogenesis due to their importance in intracellular trafficking, as well as maintaining lipid homeostasis. Overall, this work identifies Nus1 and Rer2 as important regulators of *C. albicans* filamentation and highlights the power of functional genomic screens in advancing our understanding of gene function in human fungal pathogens.

## Introduction

Fungal pathogens infect billions of individuals globally and are responsible for 2.5 million deaths annually (Brown *et al*. 2012; Fisher *et al*. 2020; Denning 2024). Despite these staggering numbers, only a small subset of fungal species are capable of causing life-threatening infections in humans, with species belonging to *Candida*, *Cryptococcus*, *Aspergillus*, and *Pneumocystis* genera accounting for greater than 90% of all invasive mycoses (Brown *et al*. 2012; Fisher *et al*. 2020). Within the *Candida* genus, *Candida albicans* is the leading cause of systemic candidiasis with a ∼40% mortality rate, despite therapeutic intervention (Pfaller *et al*. 2019; Fisher *et al*. 2020). As an opportunistic fungal pathogen, *C. albicans* is an asymptomatic colonizer of mucosal surfaces of approximately 80% of healthy individuals (Romo and Kumamoto 2020). However, it is also a primary etiological agent of mucosal infections of the oral and genital tracts, and the cause of life-threatening systemic infections in individuals who have compromised immunity or have endured invasive clinical procedures (Pfaller *et al*. 2019; Fisher *et al*. 2020). Thus, understanding the factors that contribute to *C. albicans* pathogenicity is critical to improving human health.

The success of *C. albicans* as a pathogen can be attributed to a suite of virulence factors, including its ability to transition between yeast and filamentous morphologies, which is tightly regulated (Noble *et al*. 2017). Filamentous growth in *C. albicans* is triggered by multiple host-relevant cues, including serum, nutrient limitation, alkaline pH, carbon dioxide, and elevated temperature, and *C. albicans* relies on complex and interconnected signaling pathways to regulate morphogenesis in response to these stimuli (Shapiro *et al*. 2011; Chow *et al*. 2021). Two of the major signaling pathways that control filamentation are the cyclic AMP (cAMP)-protein kinase A (PKA) pathway and the Cek1 mitogen-activated protein kinase (Cek1-MAPK) pathway (Shapiro *et al*. 2011). While many studies have examined the signaling cascades that control *C. albicans* morphogenesis, very few studies have provided a large-scale systematic assessment of the genes and pathways that regulate this important virulence trait in response host-relevant conditions (Noble *et al*. 2010; Chauvel *et al*. 2012; O'Meara *et al*. 2015; Hossain *et al*. 2021).

Functional genomic screens that have been performed have provided key insights into pathways that govern diverse attributes in *C. albicans*, including morphogenesis (Noble *et al*. 2010; Chauvel *et al*. 2012; O'Meara *et al*. 2015; Hossain *et al*. 2021; Case *et al*. 2023). The Gene Replacement And Conditional Expression (GRACE) collection stands out as one of the most extensive mutant libraries available for *C. albicans*. Currently, this collection is comprised of 3,193 mutants, representing approximately half of the *C. albicans* genome (Roemer *et al*. 2003; Fu *et al*. 2021). For each strain, one allele of a target gene is deleted, and the remaining allele is placed under the control of a tetracycline-repressible promoter. The addition of tetracycline, or its analogue doxycycline (DOX), represses expression of the remaining wild-type allele, enabling analysis of phenotypes associated with gene depletion and the study of essential gene function. The systematic examination of this library has contributed to the identification of essential genes, as well as genes that modulate antifungal drug susceptibility, and genes necessary for morphogenesis in response to select filament-inducing cues (O'Meara *et al*. 2015; Caplan *et al*. 2018; Fu *et al*. 2021; Hossain *et al*. 2021; Lee *et al*. 2022).

To gain further insights into the genes that govern filamentation in *C. albicans*, we adopted a functional genomic screening approach with the GRACE collection to identify genes that were required for filamentous growth in host-relevant conditions, specifically tissue culture medium supplemented with serum at 37°C with 5% CO_2_ (Case *et al*. 2023). Here, we describe complementary analyses that involved both manual annotations and machine learning to define the genes important for filamentation in this host-relevant condition. Through this analysis, we identified 307 genes required for filamentation, including known regulators of filamentation such as *FLO8* and *ENT2*, validating our approach. In addition, we identified *orf19.5963* as a novel regulator of filamentous growth, which based on sequence homology to the model yeast *Saccharomyces cerevisiae* is predicted to encode the prenyltransferase Nus1 (Skrzypek *et al*. 2017). We further highlight that Nus1 and its predicted interacting partner Rer2 are important for filamentation due to their role in regulating endocytic membrane architecture required for intracellular trafficking, as well as lipid homeostasis. Overall, this work identifies genes important for *C. albicans* morphogenesis in host-relevant conditions, describes a novel role for *NUS1* in this process, and highlights the importance of functional genomic screens in advancing our understanding of *C. albicans* biology.

## Materials and methods

### Growth conditions

*C. albicans* strains were grown under standard laboratory conditions at 30°C in yeast extract-peptone-dextrose (YPD) medium (1% yeast extract, 2% peptone, and 2% D-glucose) or RPMI medium, unless otherwise stated. Depletion of *C. albicans* target gene expression for GRACE mutants was achieved by adding the indicated concentration of DOX to the growth medium. All strains were archived in 25% glycerol in YPD medium and maintained at −80°C unless otherwise stated.

### Strain construction

To generate a *tetO-RER2/Δ* GRACE strain, the NAT cassette and tetO promoter were PCR amplified from pLC763 (Hossain *et al*. 2020) using primers containing 20–22 base pairs complementary to the promoter replacement cassette and 70 base pairs of homology upstream or downstream of the start of the gene of interest (designated primer 1: CCATATCATTTTCTTATACCACTACCCAGGTTTGTGATATATTGCAGTTGACACTTTTAGTTCTCATATTactggatggcggcgttagtatc and primer 2: TACCAAGCATTTTCTTGCAAGTTAATAACACCTGTTTATAACCTGGAAACGTAGACACCCAATCAGACATcgactatttatatttgtatg). The cassette was transformed into the *RER2/Δ* heterozygous mutant (Xu *et al*. 2007). NAT-resistant colonies were PCR tested for upstream integration of the tetO promoter using “Primer 3” (CAATAGAAATGAGTGGTGCAGC) that anneals ∼195 base pairs upstream of the gene of interest and a primer that anneals within the NAT cassette (oLC6853: CGCAGAAAGTAATATCATGC). Transformants were also genotyped using “Primer 4” (CGTATACAGTGGCACAT TTGAC) that anneals ∼247 base pairs downstream of the start codon and a primer that anneals within the tetO cassette (oLC2535: GTTTGGTTCAGCACCTTGTCG). NAT-resistant colonies were additionally tested by PCR to verify the absence of a wild-type promoter using Primer 3 and Primer 4.

### Functional genomic screen

Functional genomic screening was performed as described in Case *et al*. (2023). Images of each well were captured on an Incucyte S3 Live-Cell Analysis System (Sartorius) using 20× magnification. Two biological replicates were performed for the screen, and the filamentation phenotype of GRACE mutants was scored manually and analyzed using Candescence (Bettauer *et al.* 2022).

### Filamentation assays

To monitor *C. albicans* filamentation, cultures were grown overnight in 10 mL of YPD at 30°C. Overnight cultures were then subcultured to an OD_600_ of 0.5 in 10 mL of YPD medium with or without 0.05 μg/mL DOX and incubated at 30°C for 18 hours under shaking conditions. The following day, overnights were diluted to an OD_600_ of 0.1 in their respective medium without or with 5 μg/mL DOX. A total of 100 μL was transferred to a 96-well plate and incubated under the following static growth conditions: YPD at 30°C for 5 hours, RPMI medium supplemented with 3% heat-inactivated fetal bovine serum (HI-FBS) at 37°C with 5% CO_2_ for 5 hours, YPD supplemented with 10% HI-FBS at 37°C for 6 hours, Spider medium (1% mannitol, 1% nutrient broth, and 0.2% K_2_HPO_4_) at 37°C for 6 hours, YPD at 42°C for 6 hours, YPD with 25 mM hydroxyurea at 30°C for 5 hours, or YPD with 10 μM geldanamycin at 30°C for 9 hours. Images were captured either using the IncuCyte S3 Live-Cell Analysis System (Sartorius) using 20× magnification or using differential interference contrast (DIC) microscopy with a Zeiss Axio Imager.MI (Carl Zeiss) using 40× magnification.

For solid cues, saturated overnight cultures were diluted 10-fold in water, and 5 μL was spotted onto the appropriate agar plates. Media composition was the same as liquid inducing cues, except supplemented with 2% agar. Filamentation was assessed at 72 hours. All strains were grown at 37°C, except for the elevated temperature cue for which strains were grown in YPD medium at 42°C. Colony morphology for solid conditions was captured using a Bio-Rad ChemiDoc imaging system.

### Quantitative real-time-PCR

Strains were grown in liquid YPD overnight at 30°C with shaking. Overnights were subcultured to an OD_600_ of 0.5 in YPD in the absence or presence of 0.05 μg/mL DOX and grown for an additional overnight. The next day, strains were subcultured into the same conditions without or with 5 μg/mL DOX and grown to mid-log phase. Cells were pelleted at 2,000 *g* at 4°C, washed with cold, distilled water, and then flash frozen in liquid nitrogen before storing pellets at −80°C. Cells were lysed by bead beating with acid washed glass beads (Sigma G8772–500g) with the MiniBeadBeater-16 (BioSpec Products) 3 times for 45 s, with 1 minute on ice in between. RNA was extracted from the lysed cells using the QIAGEN RNeasy kit and DNase treated using the Invitrogen DNA-free DNA Removal Kit. Complementary DNA synthesis was performed using the iScript cDNA Synthesis Kit, utilizing both oligo (dT) and random primers for amplification. qRT-PCR was performed using the BioRad CFX-384 Real Time System in a 384-well plate, utilizing the Applied Biosystems Fast SYBR Green Master Mix with a 10 μL reaction volume. Amplification was performed with the following cycle conditions: 5°C for 3 minutes, then 95°C for 10 s, and 60°C for 30 s, for 40 cycles. The melt curve was completed with the following cycle conditions: 95°C for 10 s and 65°C for 10 s with an increase of 0.5°C per cycle up to 95°C. Reactions were performed in technical triplicate, and data were analyzed using the BioRad CFX Maestro software. Statistical significance was determined using a 2-way analysis of variance with Bonferroni correction for multiple comparisons with GraphPad Prism. The primer sequences used for monitoring expression of *ACT1*, *NUS1*, and *RER2* are as follows: GACCTTGAGATACCCAATTG/CAGCTTGAATGGAAACGTAG, GCAATGCAGTTGACTTAGAG/GTTCTGGTCCCACTAATTC, and GGACGGAAACAGAAGATACGC/GCGTATACAGTGGCACATTTGAC.

### Fluorescence microscopy

Overnight cultures grown in YPD were subcultured to an OD_600_ of 0.2 in YPD without or with 5 μg/mL DOX. Cultures were grown at 30°C for 3 hours with shaking at 200 rpm. To visualize endocytic membranes, cells were stained with 8 μM FM4-64 (Thermo Fisher Scientific, # T3166) in the dark for 30 minutes at 30°C with shaking. After 30 minutes, cells were resuspended in 1 mL YPD medium to remove free FM4-64 and added to 4 mL of fresh YPD medium. Cells were incubated in FM4-64-free medium for 90 minutes at 30°C, spun down, washed 2 times in 1× PBS, and resuspended in 1× PBS. To visualize fluorescence, images were taken and visualized using the red fluorescent protein (RFP) filter on a Zeiss Axio Imager.M1 at 40× with constant exposure. To visualize lipid droplets, cells were stained with 1 μg/mL BODIPY (Cayman Chemical, 25892) for 10 minutes at 30°C and washed twice in 1× PBS before microscopy. To visualize fluorescence, images were taken and visualized using the enhanced green fluorescent protein (EGFP) filter on a Zeiss Axio Imager.M1 at 40× with constant exposure. To visualize cell death, cells were pelleted, washed 2 times in 1× PBS, and resuspended in PBS with 1 µg/mL propidium iodide (Sigma-Aldrich, P4170). For ethanol treatment, cells were resuspended in 70% ethanol and incubated for 30 minutes at 30°C prior to washing in PBS and staining with propidium iodide. Cells were imaged at 40× using DIC microscopy and using the dsRed channel on a Zeiss Axio Imager.MI microscope (Carl Zeiss) at the same exposure time.

## Results

### Functional genomic screen identifies mutants unable to filament in response to a host-relevant condition

**Fig. 1.**
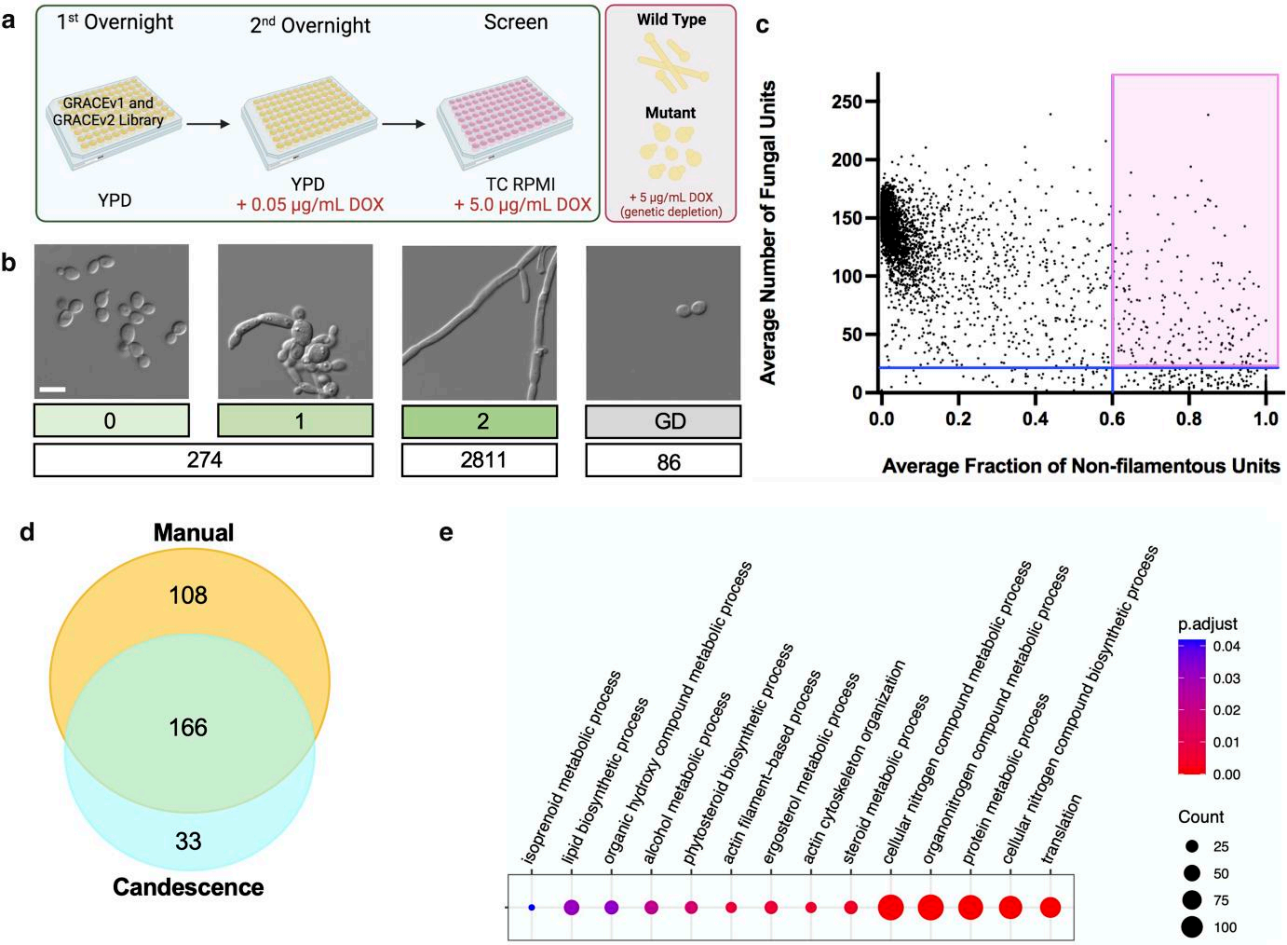
Functional genomic screen identifies mutants unable to filament in response to a host-relevant condition. a) The GRACE library (both GRACEv1 (Roemer *et al*. 2003) and GRACEv2 (Fu *et al*. 2021)) was cultured overnight in 96-well plates in the presence of a low concentration of doxycycline (DOX, 0.05 µg/mL). Cells were transferred to RPMI medium supplemented with 3% heat-inactivated fetal bovine serum and a high concentration of DOX (5.0 µg/mL). Cultures were incubated under 5% CO_2_ at 37°C for 4 hours before imaging using an IncuCyte S3 Live-Cell Analysis System. The screen was performed in duplicate. b) The filamentation phenotype of each GRACE strain was manually scored on a scale from 0 to 2. Images show representative mutants that were classified into each category. Numbers below indicate number of strains that were annotated to each category by manual scoring. Scale bar represents 20 µm. c) Plot depicting the average fraction of nonfilamentous units (*x*-axis) per image vs the average number of fungal units per image (*y*-axis). Averages represent values across biological replicates. The vertical line marks the 93rd quantile, with hits defined as those strains that lie to the right of this line. The horizontal line marks the 95th quantile of all strains manually labeled as having a growth defect. The shaded quadrant indicates strains impaired in their ability to filament in response to tissue culture conditions, as determined by Candescence. d) Venn diagram depicting hits determined by manual and computational annotation of functional genomic screen images. e) Comparative gene ontology (GO) enrichment analysis carried out for the hits identified through both manual and Candescence scoring. Representative GO terms shown. Dot size indicates the number of genes associated with a given GO term and color indicates the significance of each term.

Functional genomic analyses have been used to identify genes that are involved in *C. albicans* filamentation in response to diverse morphogenetic cues, including serum and impairment of Hsp90 function (O'Meara *et al*. 2015; Hossain *et al*. 2021). To further expand upon these foundational analyses and to identify additional genes with roles in regulating filamentous growth, we leveraged the GRACE collection to identify mutants that are unable to filament in response to tissue culture conditions (Case *et al*. 2023). For this analysis, each GRACE strain was grown overnight in a 96-well plate supplemented with a low concentration of DOX (0.05 µg/mL) to initiate gene repression. From there, strains were transferred into RPMI medium supplemented with 3% fetal bovine serum and 5 µg/mL DOX, then incubated at 37°C with 5% CO_2_. These conditions included several host-relevant inducing cues including serum, a component of human blood, physiological temperature and carbon dioxide concentration, and nutrient-limited medium. After 4 hours of growth, mutants were imaged and their filamentation phenotype was examined (Fig. 1a). The entire screen was performed in duplicate to ensure rigor and reproducibility.

To identify mutants that were impaired in their ability to filament, we first scored all microscopy images visually for their degree of filamentation and assigned each image a qualitative score on a scale from 0 to 2. Specifically, a score of 0 was given to GRACE mutants that were completely blocked in filamentation (growing as yeast), a score of 1 was given to mutants with an intermediate block in filamentation (truncated filaments relative to wild type), a score of 2 was given to mutants that showed a filamentation phenotype comparable to wild-type control strains, and growth defect was assigned to mutants that did not have enough cells in the field of view to confidently call a phenotype (Fig. 1b). Through the manual method, mutants were considered as hits if they were scored as 0 or 1 in both replicates of the screen. As a complementary approach, we also leveraged a deep learning-based method that automatically detects individual *C. albicans* cells within a microscopy image and labels them based on their morphology through a method termed Candescence (Bettauer *et al*. 2022; Case *et al*. 2023). The Candescence labeling system is similar to the manual method, but rather than giving an overall score to an image, Candescence individually labels every fungal cell in an image based on the degree of filamentation. With Candescence, a score of 0 corresponds to *C. albicans* cells in the yeast state, 1 corresponds to cells that exhibit the first stages of filamentation, 2 corresponds to cells exhibiting wild-type levels of filamentation, and 3 corresponds to irresolvable clusters of overlapping filamentous cells. To define hits using Candescence, we determined the average fraction of nonfilamentous cells for each mutant by calculating the proportion of *C. albicans* cells labeled as nonfilamentous (0 or 1) relative to the total number of fungal cells, averaged across the 2 replicates (Supplementary Table 1A). For the deep learning-based approach, mutants blocked in filamentation (hits) were considered as those with a minimum average of 21 cells per image, corresponding to the 95th percentile of strains labeled manually as growth defect (Fig. 1c, horizontal line), and with an average fraction of nonfilamentous cells exceeding 59.5% (Fig. 1c, vertical line, 93rd quantile).

Using both manual and Candescence scores, 307 genes were identified to be important for *C. albicans* filamentation in response to tissue culture conditions (Supplementary Table 1B). A total of 166 of the 307 genes were identified by both manual scoring and Candescence, while 108 of the 307 genes were identified to be important for filamentation based on manual scoring alone and 33 out of 307 genes were identified through the computational method alone (Fig. 1d). Discrepancy between the genes identified through each approach is due in part to the inherent differences in methodologies of assigning scores. Candescence was better capable of identifying hits from densely populated images. For example, for strains with an average number of fungal cells per image of greater than 80, Candescence identified 13 GRACE strains with an average fraction of nonfilamentous cells exceeding 59.5% that were not identified as a hit by manual scoring. However, the manual method proved better at identifying hits when there were few fungal cells per image, as this approach identified 47 GRACE strains that were blocked in filamentation but had less than 21 fungal cells per image and were therefore excluded by Candescence. This highlights the usefulness of each method in identifying genes important for filamentation.

To focus on the most robust hits, comparative gene ontology (GO, biological process) enrichment analysis was carried out for those hits identified through both manual and/or Candescence approaches (Fig. 1e and Supplementary Fig. 1). Two of the most significantly enriched biological processes identified amongst the overlapping hits were actin cytoskeleton organization and ergosterol metabolic process, categories previously implicated in filamentous growth (O'Meara *et al*. 2015). In addition, protein metabolic process and isoprenoid metabolic process were significantly enriched with genes important for filamentation identified through both analyses. One such gene within the latter category is the uncharacterized open reading frame, *orf19.5963*, which is predicted to encode the prenyltransferase Nus1 based on orthology to *S. cerevisiae* (Skrzypek *et al*. 2017).

### Nus1 and Rer2 are important mediators of filamentation in part through their role in vacuolar integrity and lipid homeostasis

**Fig. 2.**
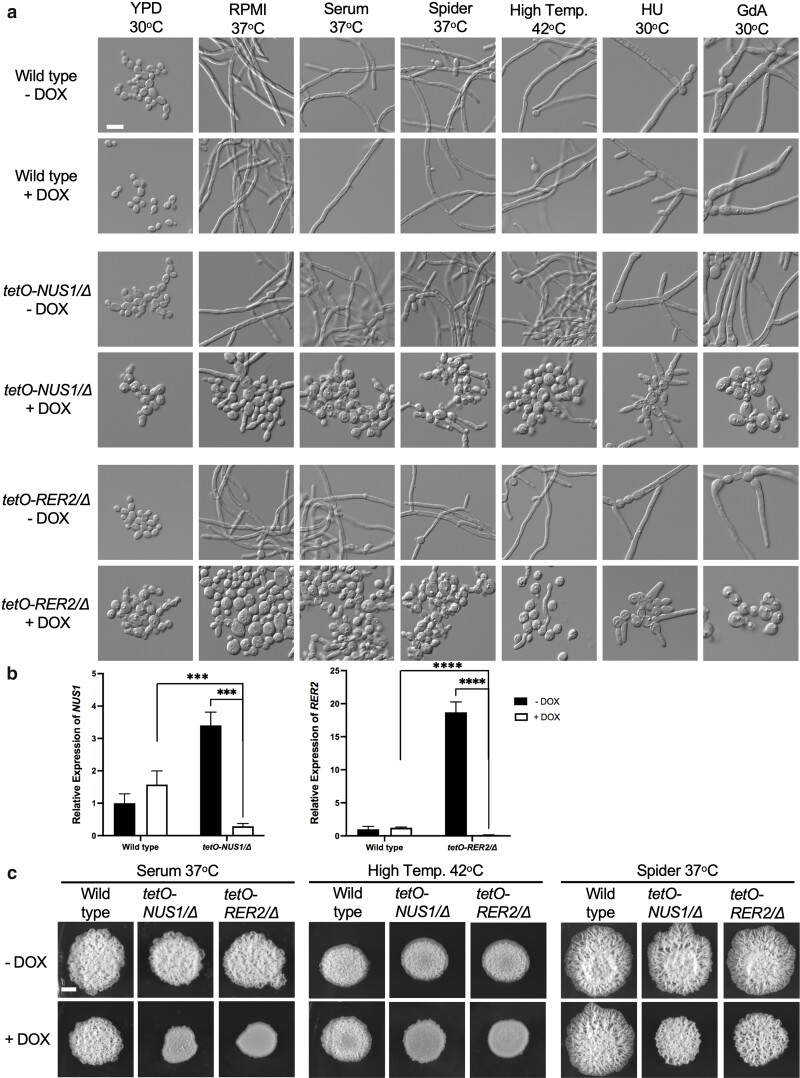
*NUS1* and *RER2* are required for filamentation in response to diverse cues. a) *NUS1* and *RER2* are required for filamentation under diverse liquid filament-inducing cues. Cells from overnight cultures were subcultured in the absence and presence of 5 μg/mL doxycycline (DOX) in static conditions in the following inducing cues: YPD at 30°C for 5 hours, RPMI supplemented with 3% heat-inactivated fetal bovine serum (HI-FBS) at 37°C with 5% CO_2_ for 5 hours, YPD with 10% HI-FBS at 37°C for 6 hours, Spider medium at 37°C for 6 hours, YPD at 42°C for 6 hours, YPD with 25 mM hydroxyurea (HU) at 30°C for 5 hours, and YPD with 10 μM geldanamycin (GdA) at 30°C for 9 hours. Scale bar represents 20 µm. b) Relative *NUS1* and *RER2* expression in wild-type, *tetO-NUS1/Δ*, and *tetO-RER2/Δ* strains in the absence and presence of 5 μg/mL DOX. Values shown are *NUS1* and *RER2* expression levels relative to *ACT1* and normalized to the wild-type strain in the absence of DOX. Error bars represent mean ± SEM of technical triplicates. Data were analyzed using an unpaired 2-tailed *t*-test; ***P-value < 0.001; ****P-value < 0.0001. c) *NUS1* and *RER2* are required for filamentation under diverse solid filament-inducing cues. Cells from overnight cultures were spotted onto plates and imaged after 72 hours of incubation in the following conditions: YPD agar with serum incubated at 37°C, YPD agar incubated at 42°C, and Spider agar incubated at 37°C. Scale bar represents 5 mm.

To further interrogate the role of *orf19.5963* in *C. albicans* filamentation (herein referred to as *NUS1*), we first wanted to confirm the phenotype observed in the primary screen, as well as assess whether Nus1 was required for filamentation in other inducing cues. A wild-type control and *tetO-NUS1*/*Δ* mutant were grown overnight in the absence and presence of DOX. The strains were then subcultured into diverse filament-inducing cues. Through microscopic analysis, we showed that *NUS1* is an important mediator of filamentation in tissue culture medium (RPMI), rich medium (YPD) supplemented with serum, Spider medium, YPD medium incubated at high temperature, YPD medium supplemented with the cell cycle inhibitor hydroxyurea, and YPD medium supplemented with the Hsp90 inhibitor geldanamycin (Fig. 2a). While these cues induced robust filamentous growth of the wild-type strain as well as the *tetO-NUS1*/*Δ* in the absence of DOX, the addition of DOX to the GRACE strain blocked filamentous growth under all conditions (Fig. 2a). This was attributed to transcriptional repression of *NUS1* as confirmed by quantitative RT-PCR, as levels of *NUS1* were significantly reduced in the *tetO-NUS1/Δ* strain in the presence of DOX relative to wild type (Fig. 2b). Additionally, we monitored the ability of the *tetO-NUS1/Δ* strain to form wrinkly colonies on solid filament-inducing agar. By spotting inoculum onto agar plates and allowing strains to grow for several days, we observed that the *tetO-NUS1/Δ* strain was defective in forming wrinkly colonies on serum-containing agar as well as agar plates incubated at elevated temperature (42°C) when DOX was present in the media despite forming robust colonies comparable to wild type (Fig. 2c). Interestingly, the *tetO-NUS1/Δ* strain formed wrinkly colonies upon growth on Spider medium agar after 72 hours of incubation in the presence of DOX, highlighting that the strain is capable of forming wrinkly colonies, a phenotype correlated with filamentous growth. Thus, *NUS1* is an important mediator of filamentous growth and wrinkly colony formation in most, but not all, inducing cues.

In *S. cerevisiae*, Nus1 forms the dehydrodolichyl diphosphate synthase complex with Rer2 (Sato *et al*. 1999; Grabinska and Palamarczyk 2002). Furthermore, *RER2* has previously been shown to be important for *C. albicans* filamentation in select inducing cues, including liquid serum medium and solid serum agar (Juchimiuk *et al*. 2014). To assess the role of *RER2* in filamentation, we generated a *tetO-RER2/Δ* strain and confirmed significant transcriptional repression of *RER2* upon incubation of the strain with DOX relative to wild type untreated (Fig. 2b). The strain was then grown in various inducing cues in the absence and presence of DOX. Interestingly, *RER2* was required for filamentation in response to all liquid filament-inducing cues (Fig. 2a) as well as wrinkly colony formation on serum-supplemented solid agar and agar incubated at elevated temperature. However, the *tetO-RER2/Δ* strain was able to form wrinkly colonies upon growth on Spider medium in the presence of DOX, similar to *tetO-NUS1/Δ* (Fig. 2c). Thus, *NUS1* and *RER2* both enable *C. albicans* morphogenesis in response to diverse inducing cues.

**Fig. 3.**
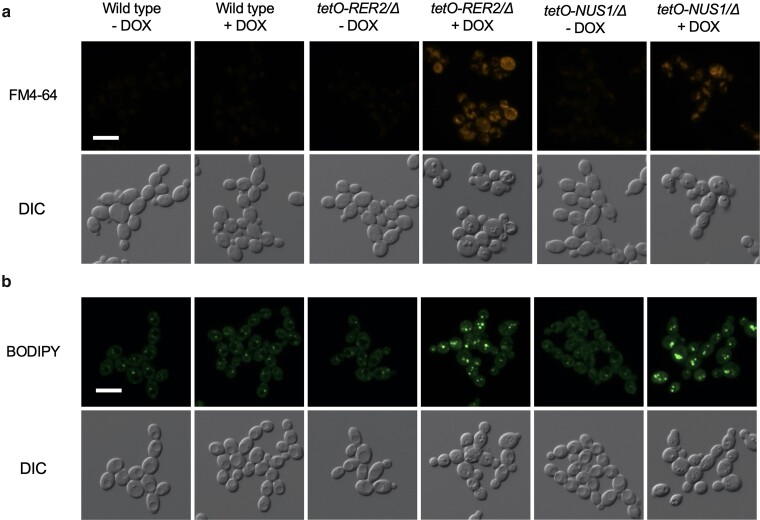
*NUS1* and *RER2* are required for intracellular membrane integrity and lipid homeostasis. Strains were grown to exponential phase at 30°C prior to staining with 8 μM FM4-64 to visualize endocytic membranes (a) or 1 μg/mL BODIPY to visualize lipid droplets (b). Scale bar represents 20 µm. All experiments were performed in biological duplicate.

Given the role of the dehydrodolichyl diphosphate synthase complex in polyprenol synthesis in both the endoplasmic reticulum and in lipid droplets (Sato *et al*. 1999; Grabinska and Palamarczyk 2002; Currie *et al*. 2014; Juchimiuk *et al*. 2014), we hypothesized that depletion of *NUS1* and *RER2* would lead to altered intracellular membrane architecture and enhanced lipid stress, which collectively have been shown to impact filamentation (Rollenhagen *et al*. 2020; Hossain *et al*. 2022). We first examined vacuolar membrane architecture of a wild-type control, as well as the *tetO-NUS1/Δ* and *tetO-RER2/Δ* strains in the absence and presence of DOX. Compared to the wild-type strain, as well as the *tetO-NUS1/Δ* and *tetO-RER2/Δ* strains in the absence of DOX, treatment of *tetO-NUS1/Δ* and *tetO-RER2/Δ* strains with DOX resulted in segmented vacuoles with heterogeneous staining of increased signal intensity (Fig. 3a and Supplementary Fig. 2b). This was not due to impaired viability, as staining with the membrane permeant dye propidium iodide led to no increase in fluorescence unlike what was observed when treated with ethanol as a control (Supplementary Fig. 2a). To determine if *NUS1* and *RER2* are important for lipid homeostasis in *C. albicans*, we also monitored lipid droplet formation. Lipid droplets are a cytosolic fat storage organelle that supports homeostasis by sequestering excess free-lipid species, providing immediate protection from lipotoxicity, and serving as critical mediators of cellular stress responses (Jarc and Petan 2019). Compared to the wild-type strain and culture conditions in the absence of DOX, the *tetO-NUS1/Δ* and *tetO-RER2/Δ* strains in the presence of DOX led to a dramatic induction of lipid droplet formation as monitored by BODIPY 493/503 staining (Fig. 3b). Thus, Nus1 and Rer2 are regulators of filamentation likely due to their importance in maintaining lipid homeostasis and proper endocytic membrane architecture, which is imperative for the intracellular trafficking that mediates filamentation (Chow *et al*. 2021).

## Discussion

The capacity of *C. albicans* to transition between yeast and filamentous forms is a key virulence trait that enables disease. In this study, we analyzed results from a functional genomic screen with the *C. albicans* GRACE collection (Case *et al*. 2023) to identify genes required for filamentation in response to a host-relevant condition that encompassed physiological temperature and carbon dioxide concentration, nutrient-limited medium, and serum, a key component of human blood. Through manual and machine learning image analyses, we identified 307 genes required for filamentation, including the previously uncharacterized gene *orf19.5963* (*NUS1*). As a predicted interacting partner of the cis-prenyltransferase Rer2, we confirmed that both Rer2 and Nus1 were required for *C. albicans* filamentation in response to diverse inducing cues in liquid and solid media. Interestingly, neither protein was required for wrinkly colony formation on solid Spider agar, despite being imperative for filamentation in liquid Spider medium, providing a further example of how solid vs liquid conditions often require distinct genetic circuitries to regulate filamentation (Azadmanesh *et al*. 2017; Lee *et al*. 2023). Overall, this work highlights the continued importance of leveraging functional genomic screens to identify and characterize gene function in fungal pathogens and motivates the continued expansion of genetic mutant collections to genome coverage to enable a comprehensive understanding of the factors in *C. albicans* that contribute to its virulence.

The GRACE collection used in this work is particularly powerful for the study of both nonessential genes and essential genes, as gene expression is inversely dependent on the concentration of DOX added to the culture. While high concentrations of DOX result in significant growth defects of the *tetO-NUS1/Δ* and *tetO-RER2/Δ* strains (Fu *et al*. 2021), the concentrations of DOX used in our analyses allowed us to observe the importance of Nus1 and Rer2 in *C. albicans* filamentation as these conditions still enabled growth of the strains. Another interesting facet of this collection is that many target genes are overexpressed in the absence of DOX due to the strong tetO promoter. Previous work has taken advantage of this trait to identify genes that are important for filamentation upon overexpression (Lee *et al*. 2023). Given the several advantages of the GRACE collection in advancing our understanding of *C. albicans* biology and pathogenicity, it is important to continue to expand the collection to obtain genome coverage.

In eukaryotes, cis-prenyltransferases catalyze the first step of the mevalonate pathway required for the synthesis of dolichol, which acts as the prenyl lipid carrier of the high mannose oligosaccharide chain in protein N-glycosylation (Grabinska and Palamarczyk 2002). Phosphorylation of dolichol to produce dolichyl phosphate also occurs in the O-mannosylation pathway, which is essential for cell wall integrity in *S. cerevisiae* (Strahl-Bolsinger *et al*. 1999). In addition, *S. cerevisiae rer2* mutants are sensitive to hygromycin B and heat stress, accumulate aberrant ER and Golgi membranes, and are unable to undergo invasive growth (Sato *et al*. 1999; Grabinska and Palamarczyk 2002; Jin *et al*. 2008). Despite this extensive characterization in the model yeast, our understanding of the phenotypic consequences of perturbing cis-prenyltransferase activity in *C. albicans* remains limited, with one study reporting that genetic impairment of *RER2* leads to defective filamentation in liquid and solid media supplemented with serum (Juchimiuk *et al*. 2014). Here, we demonstrate that *NUS1* and *RER2* are required for *C. albicans* filamentation in response to multiple inducing cues. Given the important role of the dehydrodolichyl diphosphate synthase complex at both the ER membrane and within lipid droplets in *S. cerevisiae* (Sato *et al*. 1999; Currie *et al*. 2014), we investigated the impact of genetically perturbing both *NUS1* and *RER2* on endocytic membrane architecture and lipid droplet accumulation. When these genes were repressed, it dramatically impaired intracellular membrane integrity and resulted in an accumulation of lipid droplets; phenotypes indicative of defective intracellular trafficking and aberrant membrane homeostasis. Both of these cellular processes in addition to N-linked glycosylation are critical for polarized growth in *C. albicans* (O'Meara *et al*. 2015; Hossain *et al*. 2022) providing mechanistic insight into why *NUS1* and *RER2* mutants are defective in filamentation. Given the limited arsenal of antifungal drugs and the rising prevalence of drug resistance among fungal pathogens, the identification of genes involved in virulence attributes has the potential to expand the therapeutic target space for anti-virulence therapeutic development (Gauwerky *et al*. 2009). This work also advances our understanding of the complex cellular circuitry that regulates filamentation in this important human fungal pathogen and unveils the function of a previously uncharacterized gene with implications in *C. albicans* virulence.

## Supplementary Material

jkae124_Supplementary_Data

## Data Availability

Strains and plasmids are available upon request. The authors affirm that all data necessary for confirming the conclusions of the article are present within the article, figures, and tables. Supplemental material available at G3 online.
