# Supplementary material for: The putative prenyltransferase Nus1 is required for filamentation in the human fungal pathogen Candida albicans
Source: G3 (Bethesda). 2024 Jun 14;14(8):jkae124. doi: 10.1093/g3journal/jkae124 (PMC11304969; doi:10.1093/g3journal/jkae124)
Supplement: jkae124_Supplementary_Data [file jkae124_supplementary_data.zip › Supplemental_Figure_Legends_G3-2024-405140.docx]

**Figure S1:** Dot plot representing GO Process enrichment results for the 307 genes identified as being important for filamentation through either manual or Candescence scoring (both), the 199 genes identified as being important for filamentation through Candescence (candescence_all), the 274 genes identified as being important for filamentation by manual scoring (manual_all), or the 108 genes that were only identified as important for filamentation only through manual scoring (manual_only). No significant GO terms were identified for the 33 genes only identified as important for filamentation by Candescence. Adjusted p-value is shown on the colour scale with red being the most significant. The size of the dot corresponds to the number of genes identified in that particular GO term.

**Figure S2: *NUS1* and *RER2* are required for intracellular membrane integrity.** Strains were grown to exponential phase at 30 °C prior to staining with propidium iodide to visualize dead cells (**A**) or FM4-64 to visualize endocytic membranes (**B**). Scale bar represents 20 µm. All experiments were performed in biological duplicate.
