# Supplementary figures and images for: The putative prenyltransferase Nus1 is required for filamentation in the human fungal pathogen Candida albicans
Source: G3 (Bethesda). 2024 Jun 14;14(8):jkae124. doi: 10.1093/g3journal/jkae124 (PMC11304969; doi:10.1093/g3journal/jkae124)

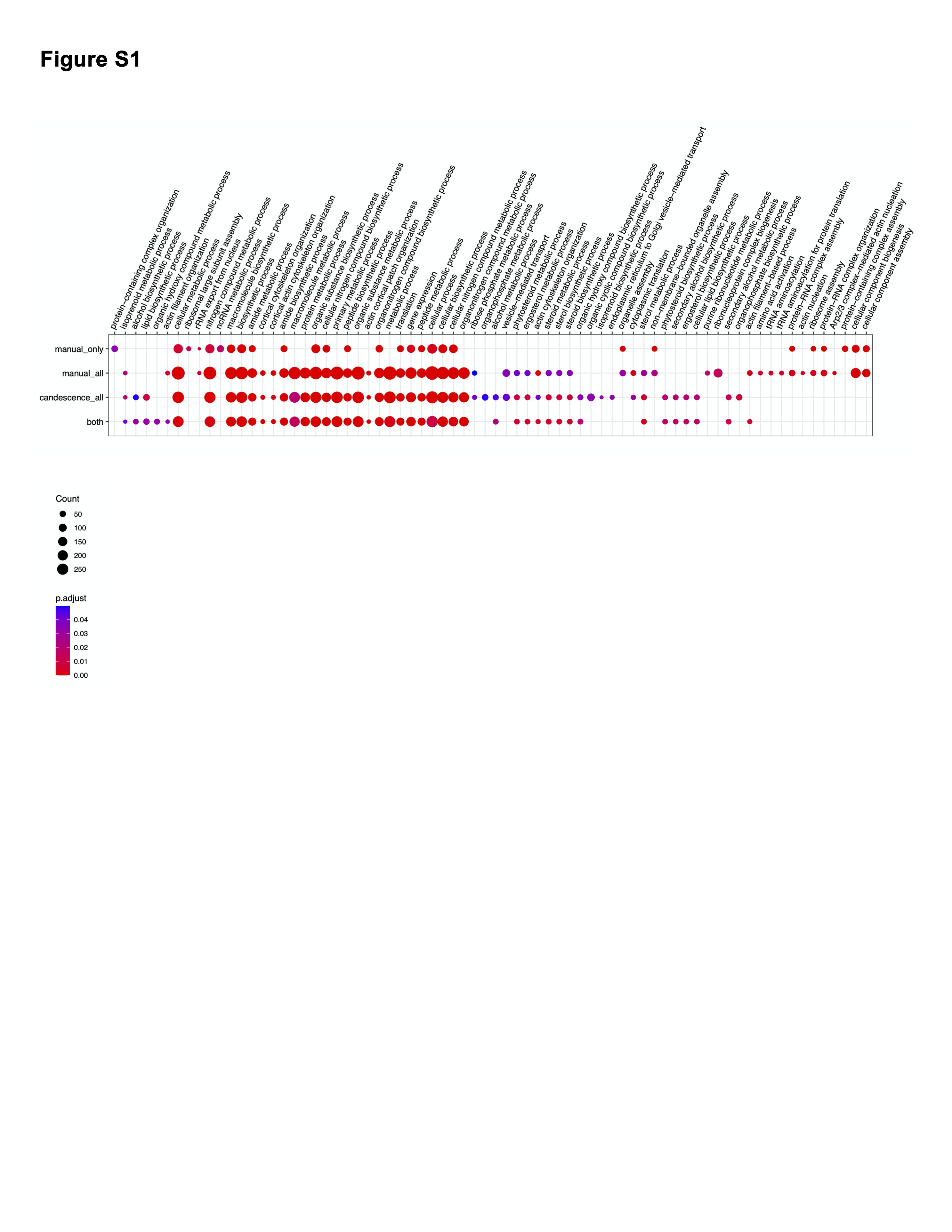

Supplement: jkae124_Supplementary_Data [file jkae124_supplementary_data.zip › Figure_S1_G3-2024-405140.tif]

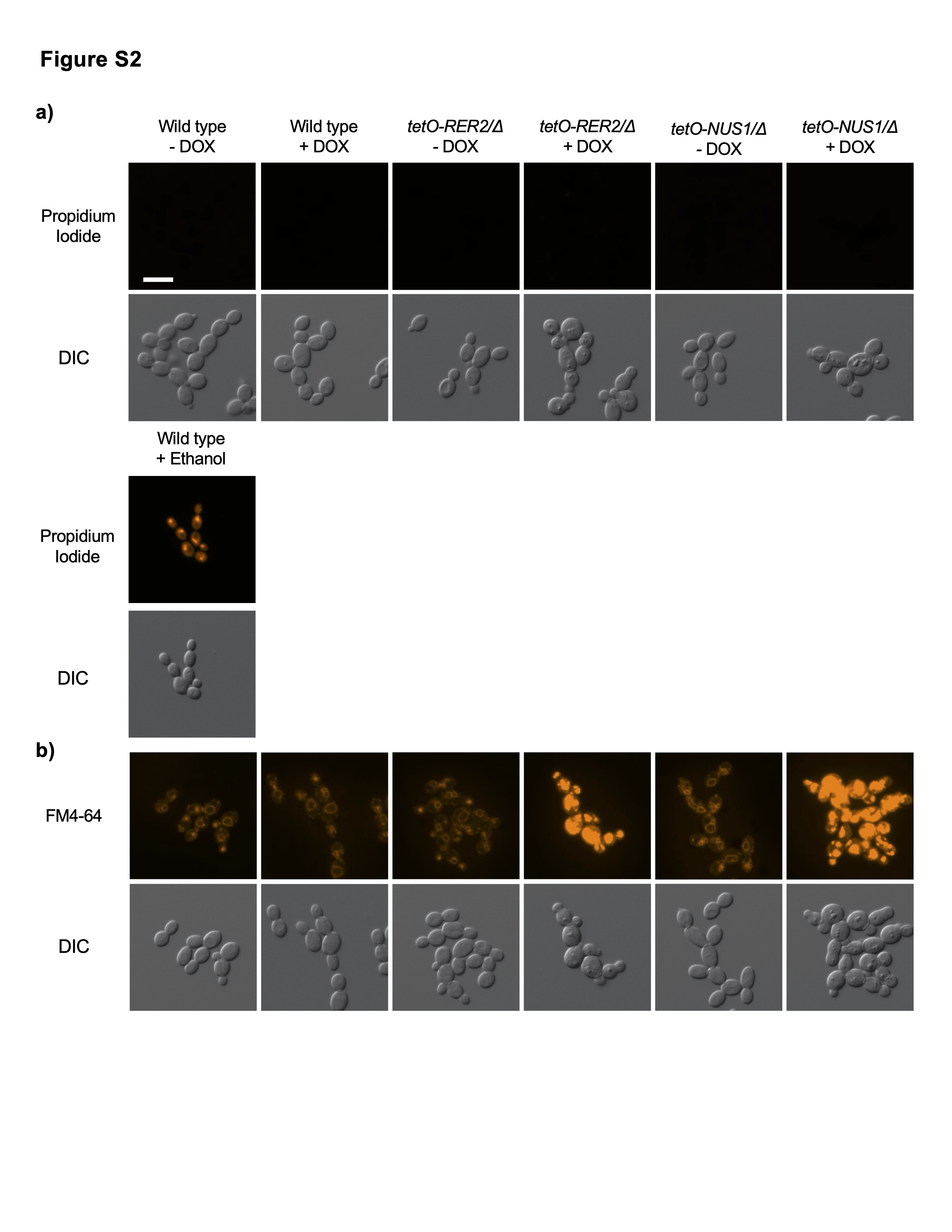

Supplement: jkae124_Supplementary_Data [file jkae124_supplementary_data.zip › Figure_S2_G3-2024-405140.tif]
